# Breast cancer in women younger than 30 years: prevalence rate and imaging findings in a symptomatic population

**DOI:** 10.11604/pamj.2014.19.35.2849

**Published:** 2014-09-16

**Authors:** Dorothy Makanjuola, Abduulmohsen Alkushi, Manal Alzaid, Omalkhair Abukhair, Fatina Al Tahan, Abdulrahman Alhadab

**Affiliations:** 1 Department of Medical Imaging King Abdulaziz Medical City for National Guard, PO Box 22490 Riyadh, Saudi Arabia; 2 Department of Pathology, King Abdulaziz Medical City for National Guard, Riyadh, Saudi Arabia; 3 Department of Surgery, King Abdulaziz Medical City for National Guard, Riyadh, Saudi Arabia; 4 Department of Oncology, King Abdulaziz Medical City for National Guard, Riyadh, Saudi Arabia; 5 Department of Radiation Oncology, King Abdulaziz Medical City for National Guard, Riyadh, Saudi Arabia

**Keywords:** Breast cancer, prevalence, imaging, Arab women younger than 30 years

## Abstract

**Introduction:**

To identify the prevalence rate of primary breast cancer in women younger than 30 years of age in a symptomatic population in Riyadh, Kingdom of Saudi Arabia. To analyze the imaging pattern and possible risk factors in cases with cancer. Breast cancer in this age group is generally rare and not clearly understood.

**Methods:**

At King Abdulaziz Medical City for National Guard, Riyadh, a retrospective 5-year (January 2006 to December 2010) data was collected from the Medical Imaging departmental records on breast imaging. Patients younger than 30 years of age were identified including those with breast cancer. The clinical presentation, risk factors, imaging findings and final outcomes were analyzed in a descriptive way. The total number of patients diagnosed with primary breast cancer was recorded.

**Results:**

Seventeen out of a total of 4873 patients younger than 30 years examined had primary breast cancer constituting a rate of 3.5 per 1000 symptomatic patients. The age range was 17 to 29 with mean of 27. The total number of patients with primary breast cancer diagnosed during that period was 413 making a percentage of 4.1% (17 out of 413) in those younger than 30 years. First presentation with a palpable mass and imaging findings of unequivocal category 5 of Breast Imaging Reporting and Data System (BI-RADS) occurred in all. Eight patients had stage I and II while nine had stage III and IV cancers. Only 2 of the 17 had first-degree family history. The youngest was 17 years old.

**Conclusion:**

A prevalence rate of 3.5 per 1000 primary cancer occurred in the symptomatic population studied and 4 in every 100 primary cancer diagnosed in the unit occurred in women younger than 30 years. First presentation, low family trait and typical imaging features of malignancy was found in all cases.

## Introduction

Breast cancer is considered rare in young women below 40 years. About 5 to 6% of total breast cancer occurs in women younger than 40 years [1]. In the epidemiology of breast cancer, young women are said to differ according to race [2]. Although breast cancer is more common in Caucasian women than African Americans, in women under 35, breast cancer is more than twice as common in African American women and is diagnosed at a more advanced stage [2].

In a recent publication [3] from the UK, the incidence of breast cancer in women younger than 35 years was 1.4% and in those younger than 30 years it was 0.43%. This low incidence provides the rationale behind avoiding unnecessary breast biopsy of young patients. A published report [4] on the epidemiology of breast cancer in women in Arab countries reveals that 50% of breast cancers occur in women under 50 years of age, whereas only 25% of breast cancers occur in women under 50 years in the industrialized world. In the United States, approximately 27% of either invasive or noninvasive cancers occur in women under 50 years of age [5].

The major risk factors in breast cancer are: age, genetic predisposition and estrogen exposure. A positive family history has a relative risk of 2.6% while having multiple first-degree relatives (mother, sister, daughter) with premenopausal cancer confers a lifetime risk as high as 50%, much of this risk is associated with a genetic defect in BRCA1 or BRCA2 [6–9]. Also, increased breast density both increases the chance of missing a breast cancer as well as increasing the absolute risk for breast cancer. Exposure to radiation, e.g., radiation therapy to the chest, is a risk factor for secondary breast cancer in adolescents [10, 11]. With the increasing awareness of breast cancer occurrence among the general population which includes the young, our diagnostic clinics are inundated with young patients presenting with all sorts of complaints ranging from breast pain, palpable mass to breast-size asymmetry or knowledge of family friends with breast cancer.

Information on breast cancer in Arab women under 30 years of age who are normally examined with ultrasonography is scanty. We do not know the prevalence rate or proportion in relation to the total breast cancers. We do not know the proportion of missed or interval cancers nor the typical imaging features or associated risk factors. This study therefore aims at indentifying the prevalence rate and proportion of breast cancer that occurs in Arab women younger than 30 years. The imaging characteristics, risk factors, e.g., family history or previous radiation, or breast density will be analyzed in cases with breast cancer.

## Methods

Retrospective data was collected from Picture Archiving and Communication System (PACS) of the Medical Imaging Department and Breast Imaging Section of King Abdulaziz Medical City for National Guard, Riyadh over a period of 5 years (January 2006 to December 2010). All the patients who were younger than 30 years of age examined including those diagnosed with primary breast cancer at the King Abdulaziz Medical City for National Guard, Riyadh were recorded. The total number of primary breast cancers diagnosed in the unit during that period was also recorded. Inclusion criteria were all patients who had initial full breast imaging and staging in our unit with pathologically confirmed breast cancer. Exclusion criteria were patients with breast involvement of generalized lymphoma or leukemia. Furthermore, patients who had breast cancer diagnoses and had surgery elsewhere before coming to us were not included. All cases were also discussed in our regular multidisciplinary breast cancer meetings. Special ethical consent for this study was not needed due to the retrospective nature of the study. Literature search was by MEDLINE and PubMed. A detailed analysis of breast cancer cases occurring in patients younger than 30 years of age at presentation was made. These patients were not stratified. They were imaged as they came from breast surgeons, from the local Primary Care Centre and from elsewhere from other Gulf Countries. They represent the usual population we care for at the King Abdulaziz Medical City for National Guard, Riyadh. Data were analyzed using descriptive statistics.

### Imaging

Patients with palpable masses had breast ultrasound examination as the first-line imaging examination while those with clinically obvious cancers such as bulging masses and/or deformed breast outlines had mammography as the first-line imaging examination; however, subsequently all had both ultrasound and mammography. Magnetic resonance imaging (MRI) was performed in some cases to show the extent of tumor spread and possible multifocality or multicentricity or before neoadjuvant chemotherapy. Computed tomography staging and isotope imaging were also obtained before treatment.

### Ultrasound Imaging

This was performed with an UltraMark 9 Philips Medical System, Netherlands using a 12-5 MH_2_linear array transducer. Real time gray-scale and Doppler images were obtained. The images were interpreted by experienced radiologists using the Breast Imaging-Reporting Data System (BI-RADS) classification [12]. The size of the masses was measured when possible. The disease was assessed as to whole breast involvement, unifocality, multifocality, multicentricity and bilaterality. Axillary nodes were also assessed. Biopsy of the lesions was made with ultrasound guidance.

### Mammography

Mammograms were obtained with LORAD MS (Hologic Selenia, United States) equipment. Standard two-view mammography was performed in both breasts in all patients. Additional views including magnification, compression, exaggerated, craniocaudal and axillary tail views were obtained when necessary. The findings were classified according to the BI-RADS Lexicon [12]. Radiographic breast density was also defined according to BI-RADS rating i.e. 1. Almost entirely fatty; 2. Scattered fibroglandular; 3. Heterogeneously dense and in type; 4. Extremely dense breast. The normal breast of the patient was used for the classification. BI-RADS [12] rating instead of computerized grading has been used because studies using observer ratings have been shown to have similar estimates [13]. MR Imaging: Patients were examined in a prone position with breasts hanging freely in a dedicated breast coil. A 3T (Philips Equipment, Netherlands) was used.

The usual departmental protocol was followed including STIR, T2 and T1 weighted, three-dimensional (3D) fat-suppressed gradient-echo (18/4) flip and 50° bandwidth 50KH2 sequence. Pre-contrast images were obtained followed by three sequential images after bolus contrast injection of 2 mmol/kg bodyweight with Magnevist (Schering Berlin, Germany). Subtraction views and maximum intensity vascular phase (MIPS) were also obtained. Areas of abnormal breast enhancement were depicted in the subtraction views and often highlighted in the MIPS. Kinetic study was done in the dynamic sequence. Areas of abnormal enhancement were analyzed according to mass or non-mass, linear ductal and asymmetric patterns. The findings were rated according to the BI-RADS Lexicon [12].

Follow up of patients who received neoadjuvant chemotherapy were performed with ultrasound, mammography or MRI. Staging CT of the chest, abdomen and pelvis as well as radioisotope bone scans were performed in all patients with disease stage 2 and higher. The standard TNM grading system was used. Pathological analysis was performed by a breast pathologist, and included tumor size, type, excision margins, grade, and hormone receptor status HER2/neu and tumor stage.

## Results

**Figure 1:**
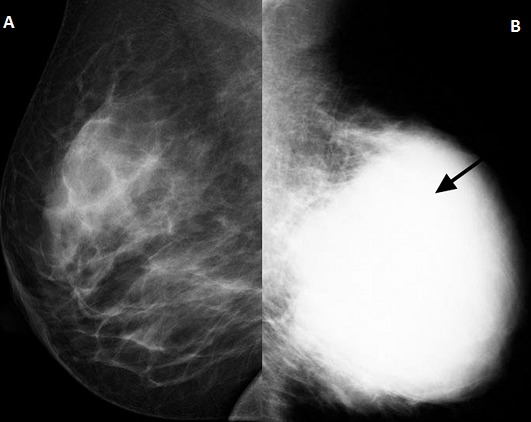
Bilateral mediolateral oblique (MLO) mammograms (age 26). A) normal breast with density category 2; B) showing advanced cancer of the left breast with a homogeneous dense mass extending to the skin (arrowed)

**Figure 2:**
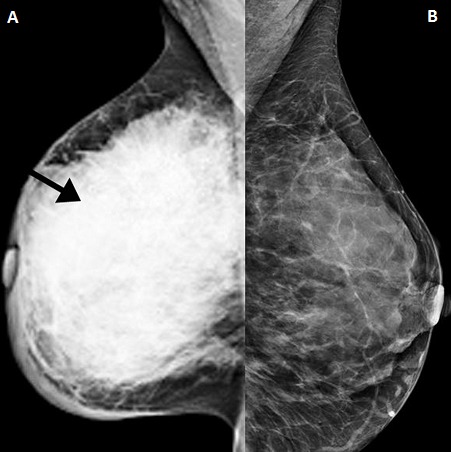
Bilateral MLO mammograms (age 28). A) showing a dense mass with advanced malignancy replacing the right breast (arrowed); B) left breast shows category 2 density

**Figure 3:**
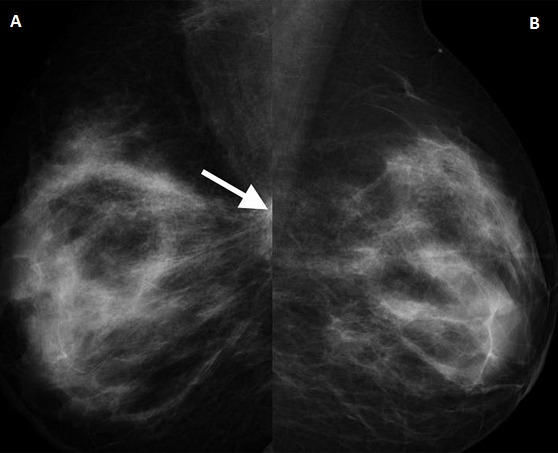
Bilateral MLO mammograms (age 29). A) showing the smallest cancer in the study. It is a deep-seated lesion with asymmetric density at the right upper quadrant (arrowed), only seen in the MLO view; B) normal breast with breast density category 3

Out of a total number of 4873 patients under 30 years old, 17 had primary breast cancers contributing 3.5 per 1000 symptomatic patients. The total number of primary breast cancers diagnosed in the study period was 413, 17 of which were younger than 30 years (4.1%). The age range was 17-29, mean 27. Notably, only one patient age was 17 i.e. below 20 years of age. In relation to family history, only 2 of the 17 patients had a first-degree (mother and sister) family history. Genetic analysis was not performed. The mammograms revealed that 13 out of the 17 had a category 2 breast density (Figure 1, Figure 2) while 4 out of 17 had a category 3 breast density (Figure 3A, B). None were in the 1 or 4 breast density category. None of the patients had previous radiotherapy, previous breast ultrasound examination or biopsy. Out of the 17 patients, 13 were parous and 3 were pregnant at the time of diagnosis.

**Figure 4:**
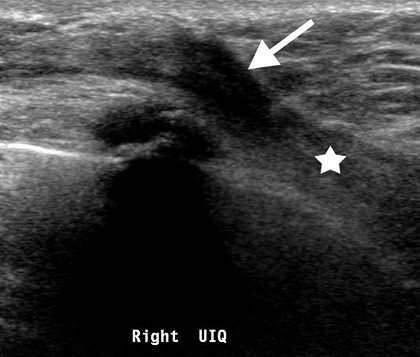
Ultrasound examination showing deep-seated angular hypoechoic lesion measuring 1.5 x 8cm (arrowed) extending to the pectoralis (starred), considered stage 4 due to pectoralis muscle involvement

**Figure 5:**
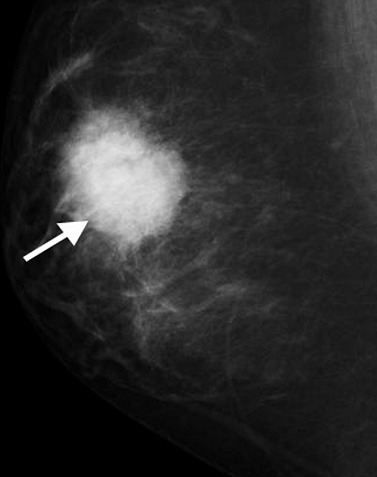
Right MLO mammography (age 17- youngest patient) showing dense speculate mass in the breast (arrowed)

**Figure 6:**
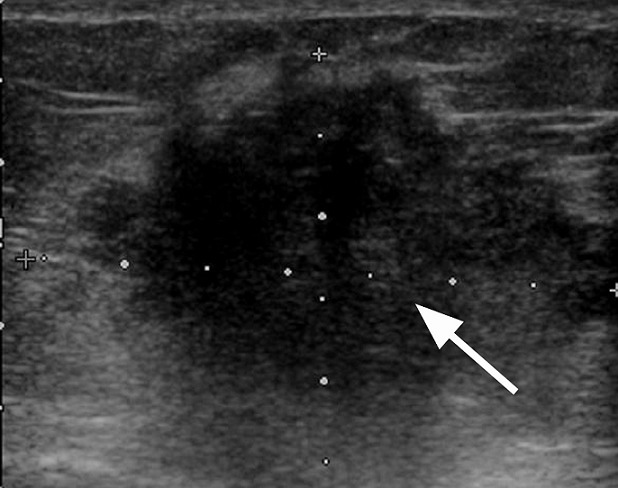
Ultrasound imaging confirms the microlobulated mass with posterior shadowing in the right breast revealing malignant characteristics (arrowed)

**Figure 7:**
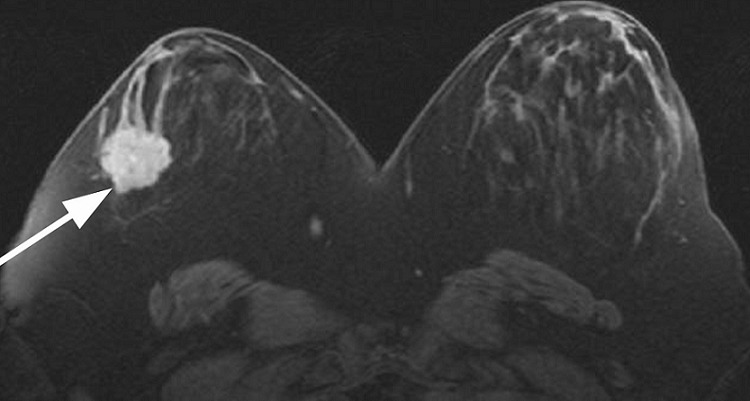
MRI subtraction maximum intensity projections (MIPs) showing solitary intensely enhancing microlobulated lesion in the right breast. Kinetic pattern also had rapid washout (not shown). Histopathology revealed secretory carcinoma

All the cases (N=17) were symptomatic and presented with palpable masses or obvious advanced unilateral changes. The lesions were identified in both ultrasound and mammograms and classified as BI-RADS category 5. Mammograms were obtained in this age group due to the suspicion of cancer. Five out of the 17 had stage 4 with near total involvement of the breast. Four had stage 3 with lesions between 5-9 cm. Stage 2 occurred in 5 cases where lesions were seen 2-5 cm. Stage 1 in 3 cases with less than 2 cm width. Figure 3A and Figure 4 show the smallest lesion in the study which was located deep in the breast. Three patients were pregnant at presentation and one of them had metastatic disease at presentation while the other two had stage 2 and 3 axillary node positive disease. Histopathological analysis revealed all had invasive ductal carcinoma except the youngest 17 years old who had secretory carcinoma (Figure 5, Figure 6, Figure 7). All except the 17-year-old patient had nuclear grade 3 tumors, 13 of the 17 had hormone receptor-negative cancers, 12 of the 17 had positive axillary node biopsy. All those with stage 1 and two of those with stage 2 had node-free disease. In the final outcome, 7 of the 17 had mastectomy, 6 patients had lumpectomy and 3 patients have expired. These three patients were those with metastatic disease identified by CT and bone scan at presentation.

## Discussion

The study reveals a prevalence rate of 3.5 per 1000 in a symptomatic population of women younger than 30 years and 4 out of every 100 breast cancer diagnosed at the breast imaging unit of King Abdulaziz Medical City for National Guard, Riyadh. It is not surprising since an earlier report [8] had shown that 50% of breast cancers occur in patients under 50 years of age in women in Arab countries.

The population studied includes patients referred from other Arab countries. Hence, the only patient who was younger than 20 (17 years) was referred from another Arab country. In an unpublished report, this 17-year-old patient is the only one we have seen in our hospital multidisciplinary team over a period of 10 years. The findings support the use of ultrasound in this age group with hardly any need for biopsy. However, biopsy is encouraged in patients over 20 years who have lesions that are not distinctly benign. It is also important to note that none of the cancer patients had previous imaging examinations and so they were not missed cancers nor interval cancers at imaging. All had BI-RADS category 5 type lesions. The distinct malignant-type morphology revealed in imaging may be a reflection of the aggressive nature of the tumor. None of the patients had previous radiation therapy.

Only 2 patients had a positive family history. They had a first-degree family history involving mother and sister. One of the two was 25 years old and had metastatic disease at presentation and has since passed away. Genetic analysis was not done and not routinely done presumably due to not being ordered by the physicians or refused by the patients. Our findings are in agreement with the study on Irish young women where patients with primary breast cancer had a low family trait [14].

Only 3 of the 17 patients had breast density of category 3. Due to the limited number of patients involved, it is difficult to draw a conclusion on the effect of density. However, these patients had breast density less than the usual pattern in young females who commonly have dense breasts which obscure findings in mammography [2]. The factors that affect breast density include age, hormone replacement therapy, menstrual cycle phase, parity, body mass index, family, and genetic tendency [6]. Out of the 17 patients, only 3 had breast density category 3. The majority of the 17 patients had breast density category 2. The relatively low density for this young population is probably related to parity and lactation since 13 out of the 17 patients were parous.

Indeed, 11 of those 13 with breast density 2 were parous. Other undocumented factors, e.g., body mass index and genetic tendency may play a role. All the cases presented with palpable masses and late presentation was common. In five of the 17 cases, the entire breast was affected clinically and was apparent in imaging. In analysis of the imagings, both advanced and smaller lesions had distinct malignant characteristics with spiculations or angular margins and microcalcification or microlobulation. The average duration of symptoms was 6 months. The possible reasons given for the late presentation in some of the patients was related to lack of knowledge with the belief that since the lesion was painless, it was not serious. The second reason was their embarrassment in complaining about disorders pertaining to the breast. Mammography was performed in these patients to achieve complete evaluation of both breasts when conservative surgery was contemplated.

Three of the 17 patients (17.6%) were pregnant at presentation. One of the pregnant patients was in the first trimester and two were in the second trimester. One had metastatic disease while the others had stage 2 and 3 diseases. This is within the reported incidence of 10-20% of breast cancer in women 30 years of age and younger [15]. Two of these patients had modified radical mastectomy and axillary lymph node dissection during pregnancy and are doing well. These two surviving cases support the current reports [14] that gestational breast cancer is not associated with a worse prognosis compared to similar staged non-pregnant breast cancer. The third patient who had metastatic disease at presentation had passed away.

The histological pattern of all except the youngest 17 years old was grade 3 invasive ductal carcinoma. The youngest patient had secretory carcinoma which is described as having a favorable prognosis [16]. It was previously called ‘juvenile carcinoma’ but now has a descriptive term ‘secretory carcinoma’ because it has distinctive features that differ from other types of ductal carcinomas such as the presence of large amounts of intracellular and extracellular secretory material [16]. Recent report has shown that metastasis and death can occur in secretory carcinoma despite the reported indolent nature of the disease and hypothesize that p63 may be a potential marker [17]. This young patient had mastectomy and axillary dissection. Like all retrospective studies, ours has its limitations. A notable limitation is the lack of genetic studies, absence of stratification of the patients in relation to origin. However, the outcomes of this study are clear. A multicentric detailed prospective study in this age group with breast cancer is suggested.

## Conclusion

In conclusion, a prevalence rate of 3.5 per 1000 symptomatic population of women younger than 30 years had primary breast cancer and 4 out of 100 primary breast cancers occurred in this group. The occurrence below 20 years of age remains very rare even in this study. First presentation with a breast mass, low family trait, and typical imaging features of malignancy with BI-RADS 5 category were found. Vigilance and awareness education to breast symptoms is required in this age group.
